# Oral ulcer treatment using human tonsil-derived mesenchymal stem cells encapsulated in trimethyl chitosan hydrogel: an animal model study

**DOI:** 10.1186/s13287-024-03694-4

**Published:** 2024-04-08

**Authors:** Hyun Seok Ryu, Celine Abueva, Andrew Padalhin, So Young Park, Seung Hyeon Yoo, Hwee Hyon Seo, Phil-Sang Chung, Seung Hoon Woo

**Affiliations:** 1 Beckman Laser Institute Korea, Dankook University College of Medicine, Cheonan, Republic of Korea; 2 Medical Laser Research Center, Dankook University College of Medicine, Cheonan, Republic of Korea; 3 School of Medical Laser, Dankook University, Cheonan, Republic of Korea; 4 Department of Otorhinolaryngology-Head and Neck Surgery, Dankook University College of Medicine, 201 Manghyang-ro, Dongnam-gu, Cheonan, 31116 Republic of Korea

**Keywords:** Tonsil-derived mesenchymal stem cell, Trimethyl chitosan, Hydrogel, Oral ulcer

## Abstract

**Background:**

Oral ulcers are a common side effect of chemotherapy and affect patients’ quality of life. While stem cell transplantation is a potential treatment for oral ulcers, its efficacy is limited as the stem cells tend to remain in the affected area for a short time. This study aims to develop a treatment for oral ulcers by using trimethyl chitosan (TMC) hydrogel with human tonsil-derived stem cells (hTMSCs) to increase the therapeutic effect of stem cells and investigate their effectiveness.

**Methods:**

Animals were divided into four experimental groups: Control, TMC hydrogel, hTMSCs, and hTMSCs loaded in TMC hydrogel (Hydrogel + hTMSCs) (each n = 8). Oral ulcers were chemically induced by anesthetizing the rats followed by injection of dilute acetic acid in the right buccal mucosa. After confirming the presence of oral ulcers in the animals, a single subcutaneous injection of 100 µL of each treatment was applied to the ulcer area. Histological analyses were performed to measure inflammatory cells, oral mucosal thickness, and fibrosis levels. The expression level of inflammatory cytokines was also measured using RT-PCR to gauge therapeutic the effect.

**Results:**

The ulcer size was significantly reduced in the TMC hydrogel + hTMSCs group compared to the control group. The stem cells in the tissue were only observed until Day 3 in the hTMSCs treated group, while the injected stem cells in the TMC Hydrogel + hTMSCs group were still present until day 7. Cytokine analysis related to the inflammatory response in the tissue confirmed that the TMC Hydrogel + hTMSCs treated group demonstrated superior wound healing compared to other experimental groups.

**Conclusion:**

This study has shown that the adhesion and viability of current stem cell therapies can be resolved by utilizing a hydrogel prepared with TMC and combining it with hTMSCs. The combined treatment can promote rapid healing of oral cavity wounds by enhancing anti-inflammatory effects and expediting wound healing. Therefore, hTMSC loaded in TMC hydrogel was the most effective wound-healing approach among all four treatment groups prolonging stem cell survival. However, further research is necessary to minimize the initial inflammatory response of biomaterials and assess the safety and long-term effects for potential clinical applications.

**Supplementary Information:**

The online version contains supplementary material available at 10.1186/s13287-024-03694-4.

## Introduction

Oral mucositis (OM), a complication of cancer treatment, commonly occurs during radiation therapy to the head and neck, chemotherapy, chemoradiotherapy, and platelet stem cell transplantation. While most cases of stomatitis caused by cancer treatment can recover quickly [1–3], stomatitis induced by medications, radiation therapy, and systemic disorders often becomes chronic, leading to treatment delays and adversely affecting cancer treatment outcomes. This issue significantly impacts the possibility of successful treatment [4].

Clinical intervention strategies for preventing OM and managing its symptoms include pain management, nutritional support, and infection prevention [5–7]. The methods employed in this approach involved standard oral care, such as using mouthwash and analgesics, have been employed but proved insufficient for chemotherapy patients [8, 9]. In the absence of effective treatments, various studies have explored the potential of cryotherapy, low-level laser treatment, and anti-inflammatory drugs [10–13]. While cryotherapy effectively reduces mucositis onset and severity, its efficacy appears limited to short-term chemotherapy [13, 14]. Low-level laser therapy (LLLT) has been recognized for its potential to reduce the incidence of stomatitis and provide therapeutic benefits. However, concerns about potential tumor activation following LLLT treatment have been raised [12, 15]. Regarding anti-inflammatory drugs, favorable outcomes have been observed in animal experiments, but there are conflicting results regarding their effects on mucositis in humans [16]. Recently, biomaterial and stem cell therapy have been considered for their potential efficacy in preventing and treating oral mucositis [17, 18].

Stem cells are specialized cells with the unique ability to differentiate into various cell types. Advancements in technology and biology have led to the exploration of different stem cell types, including embryonic stem cells, induced pluripotent stem cells (iPS), and adult stem cells such as hematopoietic stem cells, neural stem cells, and mesenchymal stem cells (MSCs)), have generated high expectations [19]. MSCs were initially predominantly isolated from bone marrow, but can also be obtained from various sources, such as fat, muscle, umbilical cord, liver, and tonsils [20–24].

Chitosan is a polysaccharide obtained through the alkaline deacetylation of chitin, a cellulose-like polymer found in animals’ cell walls, is a valuable material in the food, pharmaceutical, and medical industries due to its excellent biodegradability and biocompatibility [25, 26]. Generally, chitosan is a polymer with varying molecular weights that is insoluble in water and only dissolves in a solution of around pH 6.2. Precipitation often occurs upon the alkalinization of a chitosan solution. The inability of chitosan to maintain a solution in the physiological pH range of 7.0 to 7.4 has been a significant obstacle in using chitosan in the biological and medical fields [27]. Trimethylated chitosan (TMC), a quaternized derivative of chitosan, addresses these challenges. TMC is synthesized through the reduction methylation of amino groups by reacting chitosan under strongly alkaline conditions and methyl iodide [28]. This improves TMC’s solubility and stability in a broader pH range, including the physiological pH range, making it suitable for biological and medical applications. Modifying chitosan into TMC becomes a viable option to overcome the previously mentioned limitations. TMC is a desirable and multifunctional polymer with a positive charge due to its quaternized nature. This characteristic enables it to exhibit functions such as drug delivery, antimicrobial activity, anti-adhesive properties, hemostasis, and wound healing [29]. This study prepared hydrogels based on chitosan by incorporating glycerophosphate to create a temperature-responsive hydrogel. By adding a naturally occurring compound called beta-glycerophosphate, gelation occurs through sol-gel transition at physiological pH and 37℃. At room temperature, the hydrogel is liquid and can be injected into the body using a syringe. Upon injection, the liquid hydrogel transforms into an insoluble gel, serving as a carrier or encapsulation for drug delivery [30, 31].

Various treatments based on biomaterials, stem cells, and stem cell-derived by-products are being explored for oral mucositis. However, challenges in the oral cavity, such as saliva secretion and routine mouth movements, limit the localized administration of potential treatments (stem cells, biomaterials). Even submucosal injections face challenges with lowered retention and reduced overall efficacy. Therefore, this study focuses on increasing the therapeutic effect by overcoming said limitations by improving the retention time of and maintenance of stem cells by combining the advantages offered by thermosetting TMC hydrogel and tonsil-derived stem cells.

## Materials and methods

### Synthesis N,N,N-trimethyl chitosan(TMC)

Trimethyl chitosan (TMC) chloride was synthesized and characterized following a procedure similar to that published by Abueva et al. [28]. Low molecular weight chitosan (molecular weight of 50–190 KDa from the crustacean, Sigma-Aldrich, USA) was used to make TMC with N-methyl-2-pyrrolidone (NMP, Sigma-Aldrich, USA), sodium iodide (Merck Millipore, USA) and iodomethane (Sigma-Aldrich, USA). TMC was dialyzed for purification and then lyophilized to collect the product.

### Formulation and degradation test of thermosensitive hydrogel

**Fig. 1:**
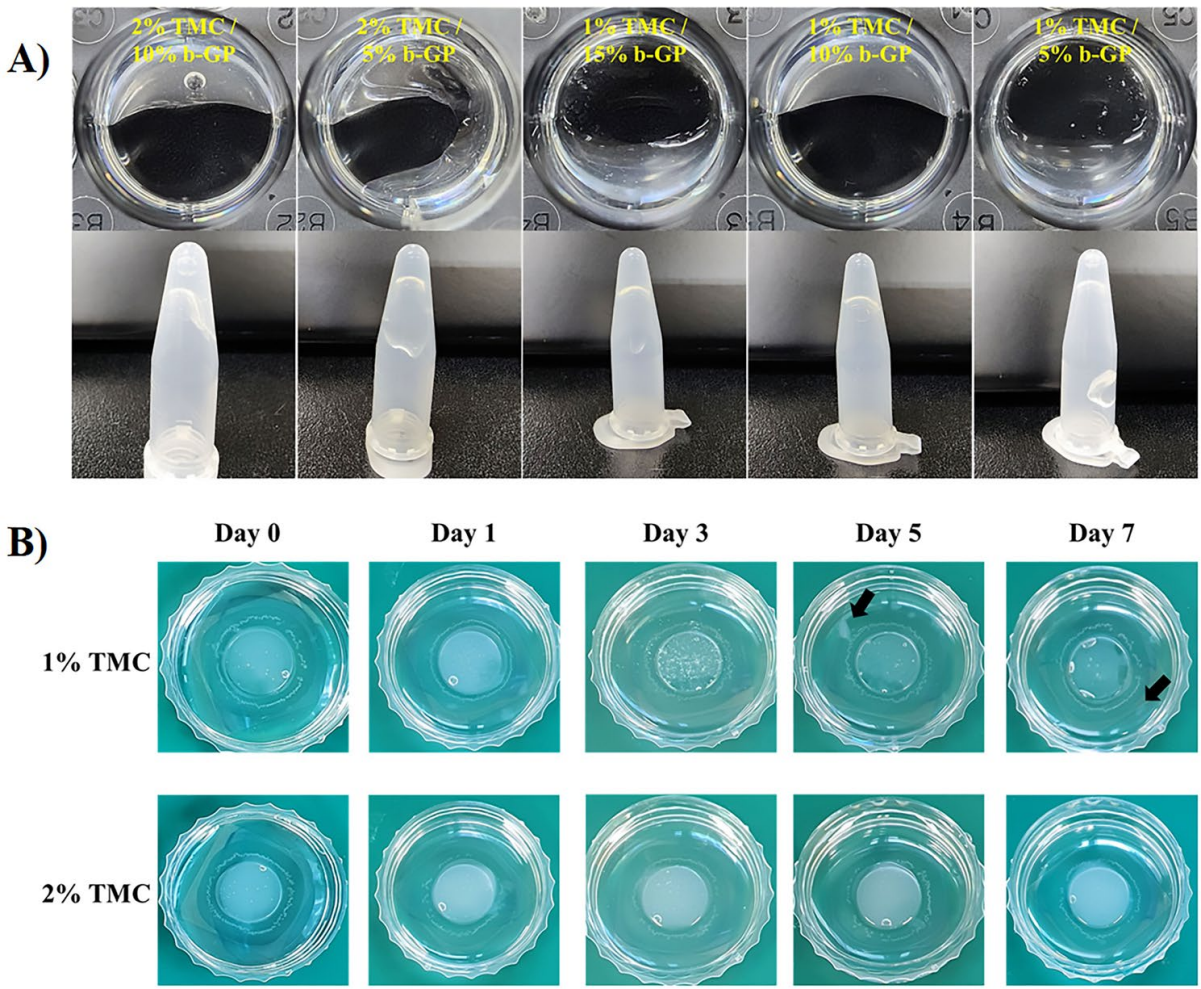
Hydrogel fabrication optimization and degradation measurement. (**A**) The images of gelled hydrogels according to the concentrations of TMC and β-gp. Gelation results were observed at 37 ℃ by mixing 1% and 2% TMC with different concentrations of β-gp. (**B**) Images of gelled samples during the degradation test observed every other day for 7 days. The arrow indicates the hydrogel fragments

A 4% (w/v) stock solution of synthesized TMC was prepared by dissolving it in Dulbecco’s phosphate-buffered saline (DPBS). Additionally, a series of β-glycerophosphate solutions (β-gp) were prepared by creating a 20% (w/v) stock solution. The chitosan solution was prepared in an ice bath on top of the stirrer and continuously mixed while adding β-gp solution dropwise. The different formulations based on TMC and β-gp composition are listed in Fig. 1A. Upon complete mixing Each solution was pipetted (400 µl) into separate wells in a 24-well plate then the plate was placed in a 37 °C incubator for 1 min. After incubation, the plate was inverted to evaluate the gelation of each of the solution. This simple test validates that the formulations that formed stable gels were able to resist flow while non-gelled solutions showed low viscosity upon inversion. Formulations that successfully solidify into a gel resisted flow upon inversion of the plate.

A degradation test was conducted by placing 500 µL of the hydrogel onto a confocal dish and allowing it to solidify. After the hydrogel solidified, 2 mL of saline was added, and the dish was placed in a 37℃ incubator. The hydrogel was visually observed daily for a period of 7 days to monitor any signs of fragmentation or dissolution.

### Isolation and characterization of human tonsil-derived mesenchymal stem cells (hTMSCs)

#### hTMSCs isolation and culture

Human tonsil-derived mesenchymal stem cell isolation and culture using discarded tonsils were obtained from children who underwent a tonsillectomy at the Dankook University Hospital (DKUH, Cheonan, Korea). We obtained written informed consent from the parents or guardians of the children who participated in the study (DKUH 2022-04-012).

The tonsil tissue was extensively washed with phosphate-buffered saline (PBS), containing 1% penicillin − streptomycin (Corning, USA), followed by digestion with collagenase type I (Sigma, St. Louis, MO, USA) for 30 min at 37 °C. The pellet was filtered through a 100 μm and 40 μm nylon mesh. Suspended cells were incubated overnight in Dulbecco’s Modified Eagle Medium (DMEM) (Corning, USA) containing 10% fetal bovine serum (FBS) (Corning, NY, USA) and 1% penicillin − streptomycin at 37 °C with 5% CO_2_. Non-adherent cells were removed by extensive washing with PBS, and adherent cells were maintained and subcultured. The cells were cultured in DMEM supplemented with 10% FBS and the media was changed every 2 days. When the TMSCs reached 80–90% confluence, they were treated with 0.25% trypsin-EDTA (Corning, USA) for 5 min. The detached cells were collected by centrifugation at 1200 rpm for 5 min.

#### Flow cytometric analysis

Flow cytometric analysis was used to characterize the phenotypes of isolated hTMSCs. At least 5 × 10^4^ cells dispersed in 100µL of PBS containing 0.5% bovine serum albumin (BSA) (37,525, Gibco) were incubated with conjugated monoclonal antibodies (PE/CD90, APC/CD105, PE/CD34, BD) for hTMSCs. Labeled cells were analyzed by flow cytometry using flow cytometry (Beckman Coulter).

### Preparation of combined treatment sample

In order to conduct a combined treatment, a composite sample containing both hydrogel and stem cells was prepared. Following the preparation of the hydrogel, the cultured stem cells were harvested and diluted in DPBS and then mixed using a stirrer. The quantity of stem cells mixed with the diluent was adjusted to be within 5% of the total volume of the hydrogel to be produced. The resulting composite treatment sample was obtained by dispersing 2 × 10^6^ cells in 100µL of the hydrogel.

### Cytotoxicity test of hTMSCs in TMC hydrogel

According to the manufacturer’s directions, the cytotoxicity in the hydrogel with hTMSCs was determined by the LIVE/DEAD™ Cell Imaging Kit (Invitrogen, USA). Briefly, cells were washed once with DPBS and then incubated with live green and dead red solutions (Live/Dead Cell Imaging Kit, Invitrogen, USA) for 15 min at room temperature in media without FBS. The cells were then imaged using EVOS with phase contrast, red fluorescence, and green fluorescence channels. image analysis was performed on EVOS M7000 (Invitrogen, USA) fluorescence micrographs (n = 10) using ImageJ software (1.53a version; National Institutes of Health).

### In vitro co-culture assay

The RAW 264.7 macrophage cells (1 × 10^6^ cells) were plated in 24 mm polyester membrane transwell inserts (0.4 μm pore size, Corning, USA) and allowed to attach for 24 h prior to treatment with lipopolysaccharide (LPS) Sigma-Aldrich, USA) for M1 phenotype induction. Macrophage cells were treated with 1 µg/mL LPS in DMEM medium supplemented with 10% FBS and 1% P/S for 24 h. The transwell inserts’ lower compartments were seeded with hTMSCs (1 × 10_6_ cells), and placed with 2 mL hydrogel or 2 mL hydrogel with hTMSCs of the same cell number the following day. Co-culture with macrophage cells was kept for 24 h in DMEM medium supplemented with 10% FBS and 1% P/S. All cultures were kept at 37oC and 5% CO2. Macrophage cells were collected the next day and prepared for RNA extraction.

### In vivo oral ulcer induction and treatment

Thirty-two male SD rats aged 7 weeks were used for this study. Rats were randomly separated into four different groups, including control (n = 8), stem cell (n = 8), hydrogel (n = 8), and combined therapy (n = 8). In each group, four animals were used per experiment for tissue analysis, and the remaining 4 animals were used for molecular biological analyses. The animals were tested after a week of adaptation, and 4 rats were housed per cage. All animals received food and water and were maintained on a 12-hour light/dark cycle in a climate-controlled animal room within the university research facility. All aspect of animal research was conducted in accordance with the guidelines of the Institutional Animal Care and Use Committee of Dankook University (DKU-23-037).

Animal modeling followed a similar procedure as previously published by Ryu et al. [31]. This study induced oral ulceration in rats by injecting 15 µL of 60% acetic acid into the right oral mucosa after anesthesia. After 3 days, the presence or absence of oral ulcers was assessed in each rat, followed by a submucosal injection of 100 µL of each respective treatment into the ulcer area. Animals in the positive control group were injected with only PBS and were designated as sham. Treatments containing stem cells (hTMSCs group and Hydrogel + hTMSCs) were prepared with 1 × 10^6^ cells. The size of the ulcer was measured using digital photos taken on Day 0, Day 1, Day 3, and Day 7.

### Histological analysis

Rats were euthanized using CO2 inhalation on Day 3 and Day 7 following the treatment, and their oral mucosa was surgically removed for subsequent histological examinations. The wound area was excised and fixed from each mucosa in a solution of 4% Paraformaldehyde. The fixed tissues were then processed, embedded in paraffin, and sliced into sections of 4 μm thicknesses. Following deparaffinization and rehydration, the prepared specimens were mounted on slides and subjected to staining with hematoxylin and eosin (H&E). The H&E staining was employed to assess the thickness of the mucosal layer and count blood vessels to evaluate wound healing. Additionally, Masson’s trichrome (MT) staining was performed to examine the collagen content and the pattern of collagen arrangement in the wound area. Using FIJI software (ImageJ with built-in plugins), the fibrous tissue composition of the regenerated tissue was quantified by performing blue color thresholding and measuring the area occupied by the segmented blue-stained collagenous tissue within the tissue area of each image [32, 33].

Immunofluorescence (IF) was performed to visualize implanted stem cell detection and immunohistochemical (IHC) staining was also performed to visualize neutrophil expression and inflammation cytokine detection. In brief, de-paraffined and rehydrated tissue sections were processed for antigen retrieval by immersion in citrate buffer (10 mM, pH 6.0) using the microwave. Tissue sections were then blocked with 3% BSA and incubated with primary antibodies, either Neutrophil elastase (1: 100; Abcam, UK), Arginase-1 (Arg-1) (1:200; Invitrogen, USA) or Inter Luekin (IL)- 1 beta (1:200; Abcam, UK) at 4 °C overnight. IF was treated Ku80(1:200; Cell signaling) and Thy-1(BD biosciences, USA) with fluorescent conjugated secondary antibody (Alexa Fluor 488, PE; Abcam, UK) and DAPI stain (nuclear stain). IHC was treated with horseradish peroxidase-conjugated secondary antibody and color-developing reagent from the EnVision Detection System Peroxidase kit (Dako Co. Denmark). Slides were then counterstained with hematoxylin to provide contrast for parts stained using the antibody. The quantitative analysis of the IHC staining was conducted using FIJI software -ImageJ with built-in plugins. High-magnification images were loaded as virtual stacks and processed with HDab color deconvolution to separate out brown-positive staining from all other colors. Thresholding was then applied, followed by particle analysis processing. The summarized values of each image were used to determine the area coverage of the brown-positive stain.

### Reverse-transcription polymerase chain reaction (RT-PCR)

Oral mucosa samples were processed by homogenizing each sample in a tube containing 1 mL of TRIzol reagent (Invitrogen, USA). The RNA extraction procedure was carried out using RNA extraction kits (Hybrid-R, Geneall, Biotechnology, Seoul, Korea), and the purity and concentration were assessed using Nanodrop 2000 (Thermo Fisher Science, USA). To generate cDNA, the purified RNA underwent synthesis using HyperScript^™^ RT Master Mix (Geneall Biotechnology, South Korea). Five genes were selected for analysis, and detailed primer information is described in Table 1. The quantification and analysis of mRNA expression were performed using the RT-PCR method employing the 7500 Real-Time PCR system (Thermo Fisher Science, USA).

The primer sequences, including the forward and reverse primers (Table 1), were designed by consulting primer-BLAST in the NCBI database and were procured from Macrogen (Macrogen, South Korea). The GAPDH gene was utilized as an internal control for normalization purposes to ensure standardized sample amounts.

**Table 1:** Primer sequences of the RT-PCR

Primer		Sequence	Note
iNOS	Forward	5ʹ- CCCTTCCGAAGTTTCTGGCAGCAG − 3ʹ	In vitro study
	Reverse	5ʹ - GGCTGTCAGAGCCTCGTGGCTTTGG − 3ʹ	
Arg-1	Forward	5ʹ - TCATCTGGGTGGATGCTCACAC − 3ʹ	
	Reverse	5ʹ - GAGAATCCTGGCACATCGGGAA − 3ʹ	
CCL5	Forward	5ʹ- CCTGCTGCTTTGCCTACATTGC − 3ʹ	
	Reverse	5ʹ- ACACACTTGGCGGTTCTTTCGG − 3ʹ	
CCL17	Forward	5ʹ- CACGCAGCTCGAGGGACCAATGTG − 3ʹ	
	Reverse	5ʹ- TCAAGACCTCTCAAGGCTTTGCAGG − 3ʹ	
GAPDH	Forward	5ʹ- AGGTCGGTGTGAACGGATTTG − 3ʹ	
	Reverse	5ʹ- TGTAGACCATGTAGTTGAGGTCA − 3ʹ	
IL-6	Forward	5ʹ- CCCTGCAGCTGGAGAGTGTGG − 3ʹ	In vivo study
	Reverse	5ʹ- TGTGCTCTGCTTGAGAGGTGCT − 3ʹ	
IL-1β	Forward	5ʹ- CCCTGAACTCAACTGTGAAATAGCA − 3ʹ	
	Reverse	5ʹ- CCCAATCAAGGGCTTGGAA − 3ʹ	
IL-10	Forward	5ʹ- TCCGGGGTGACAATAACTGC − 3ʹ	
	Reverse	5ʹ- GCAGCTGTATCCAGAGGGTC − 3ʹ	
GAPDH	Forward	5ʹ- TTCAACGGCACAGTCAAGG − 3ʹ	
	Reverse	5ʹ- CTCAGCACCAGCATCACC − 3ʹ	

### Statistical analysis

The statistical analysis and data presentation were conducted using the GraphPad Prism 8.0 software (GraphPad Software Inc., USA). The data from independent experiments were expressed as mean ± SD and statistical significance was determined using unpaired t-tests, two-way ANOVA, one-way ANOVA, and Tukey’s or Sidak’s multiple comparison post-tests.

## Results

### Optimization of hydrogel formation

To achieve optimal gelation characteristics, different concentrations of TMC (trimethyl chitosan) and β-gp (beta-glycerophosphate) were tested in various combinations for hydrogel formation with suitable gelation characteristics. Gelation testing (Fig. 1A) was performed on solutions containing either 1% or 2% TMC concentrations mixed with β-gp at 5%, 10%, and 15% concentrations. Irrespective of the TMC concentration, it was observed that the hydrogel formed most rapidly at a 10% concentration of β-gp, while the 15% concentration required the longest time for gel formation (Fig. 1A). Subsequently, a degradation test was conducted to assess the longevity of the hydrogel in an in vivo-like environment. Hydrogels solidified in a confocal dish were submerged in physiological saline and placed in a 37 °C incubator. Fragmentation and degree of degradation were visually confirmed every day for 1 week. The results revealed that the hydrogel composed of 1% TMC began to fragment and decompose on day 5 (Fig. 1B) compared to other samples. Additional data showing gradual decrease in TMC hydrogel weight can be seen in the supplementary data (S. Fig. 1.)

### Characterization of hTMSCs

**Fig. 2:**
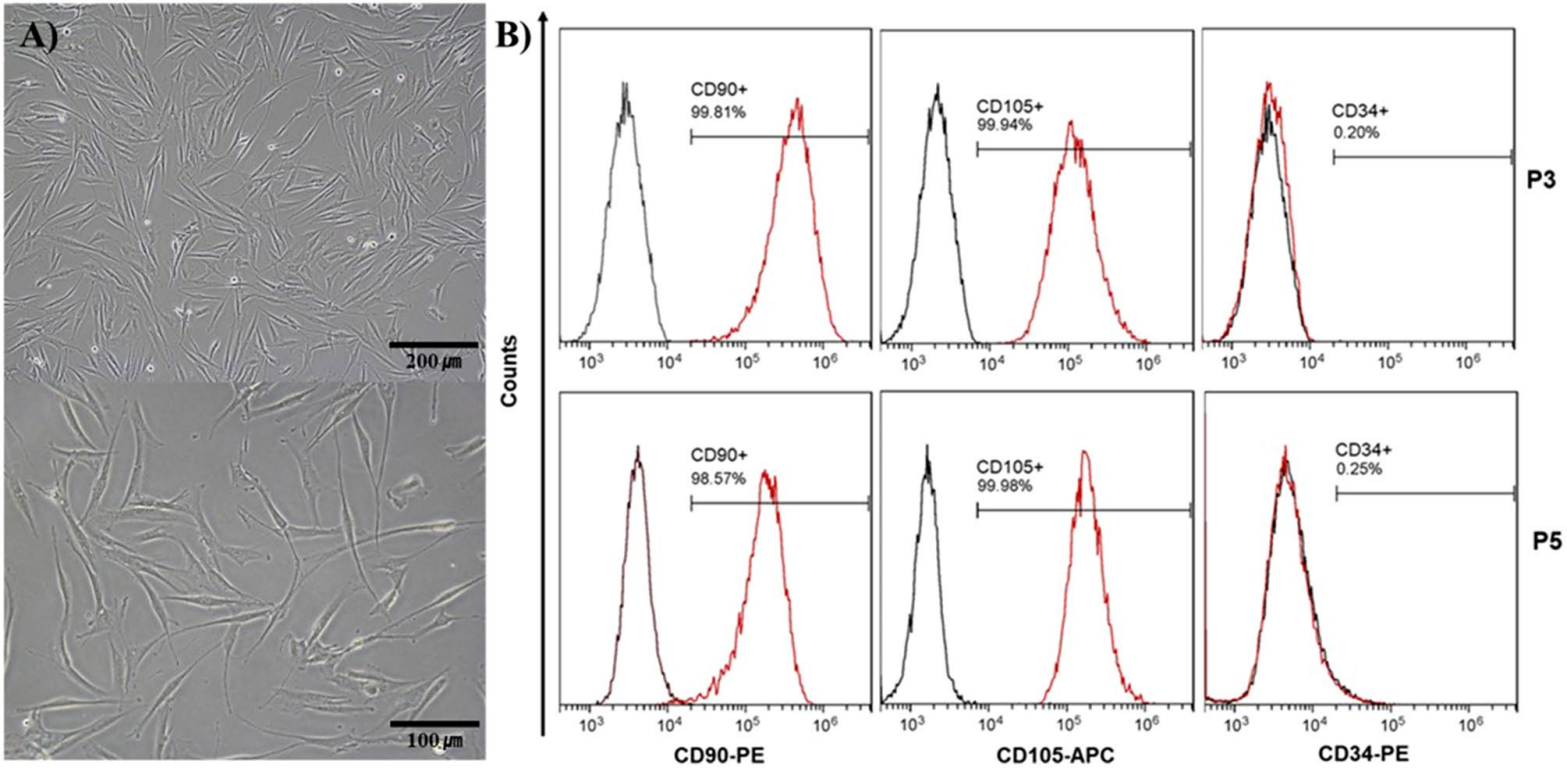
Characterization of isolated hTMSCs. (**A**) Microscopic observation image of cultured tonsil-derived mesenchymal stem cells. (**B**) Flow cytometry analysis demonstrated the presence of mesenchymal stem cell markers, including CD90 and CD105, while the hematopoietic marker CD34 was not detected in the cells

The morphology of mesenchymal stem cells isolated from tonsil tissue was observed under an optical microscope, confirming the typical form characteristic of mesenchymal stem cells (Fig. 2A). The stem cell characteristics were confirmed using four surface markers analyzed through flow cytometry (Fig. 2B). CD90 and CD105 were utilized as positive markers, while CD34 served as a negative marker. The experimental results showed that CD90 and CD105, as positive markers, matched over 98% and 99% on pages 3 and 5, respectively. Additionally, CD34, as a negative marker, was found in less than 0.25% of the cells, confirming that the cells isolated from the tonsils exhibit the characteristics of mesenchymal stem cells.

### Cell viability in TMC hydrogel

**Fig. 3:**
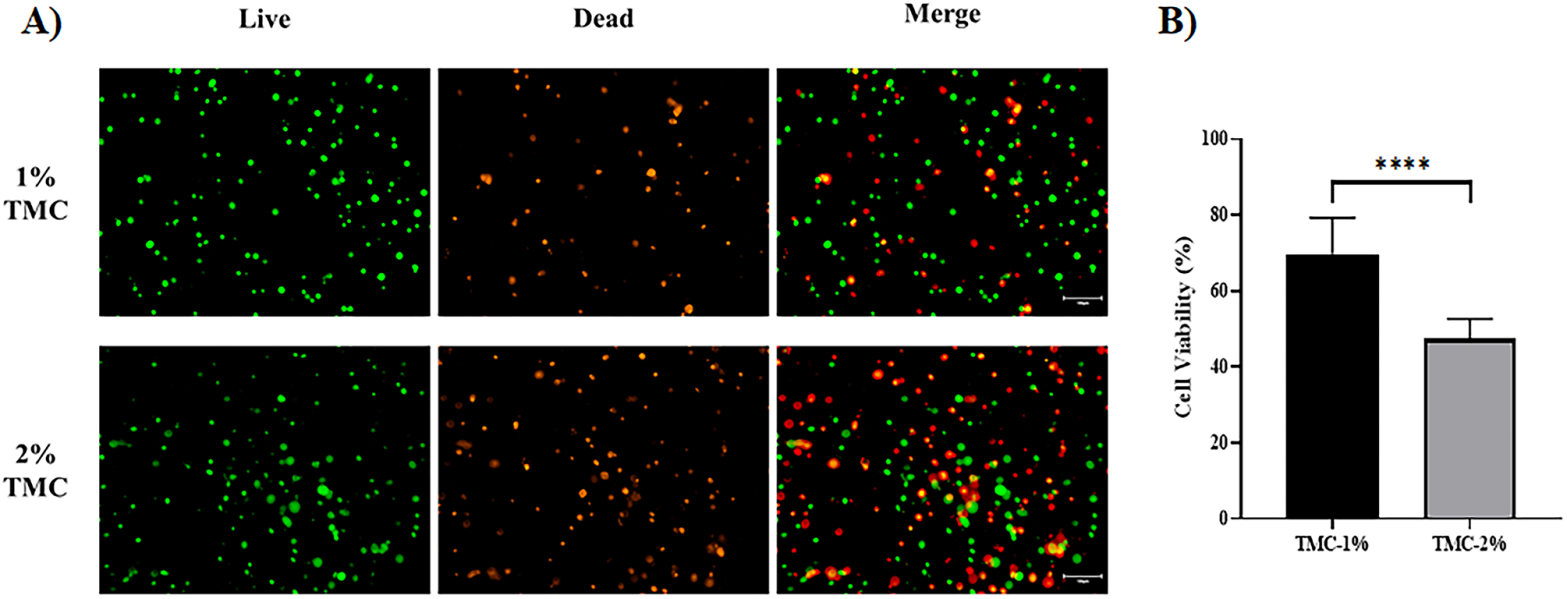
Evaluation of survival capacity of hTMSCs mixed with TMC hydrogel. (**A**) Representative live/dead cell staining images according to TMC hydrogel concentration. Fluorescence imaging using the EVOS M7000 microscope was performed on the cells. Live cells were identified by the presence of green fluorescence, whereas dead cells were distinguished by the presence of red fluorescence. (**B**) Comparison results of cell viability rate by counting survived cells per total cell. P values are indicated as ****P < 0.001 by Unpaired t-test (number of images analyzed: n = 10)

The LIVE/DEAD Viability assay was performed to visualize the stem cell viability in the hydrogel. The analysis was conducted using 1% and 2% TMC, respectively. The fluorescent imaging results showed a higher number of dead cells stained with red color in the 2% hydrogel concentration (Fig. 3A). A total of 30 images were captured, and the survival rate was determined by dividing the number of surviving cells by the total cell count. The results showed a survival rate of 69.58% ± 9.65 for the 1% hydrogel concentration, while the 2% hydrogel concentration exhibited a survival rate of 47.41% ± 5.2, indicating a 21.7% higher survival rate for the 1% hydrogel (Fig. 3B).

### Analysis of macrophage response to confirm anti-inflammatory effects

**Fig. 4:**
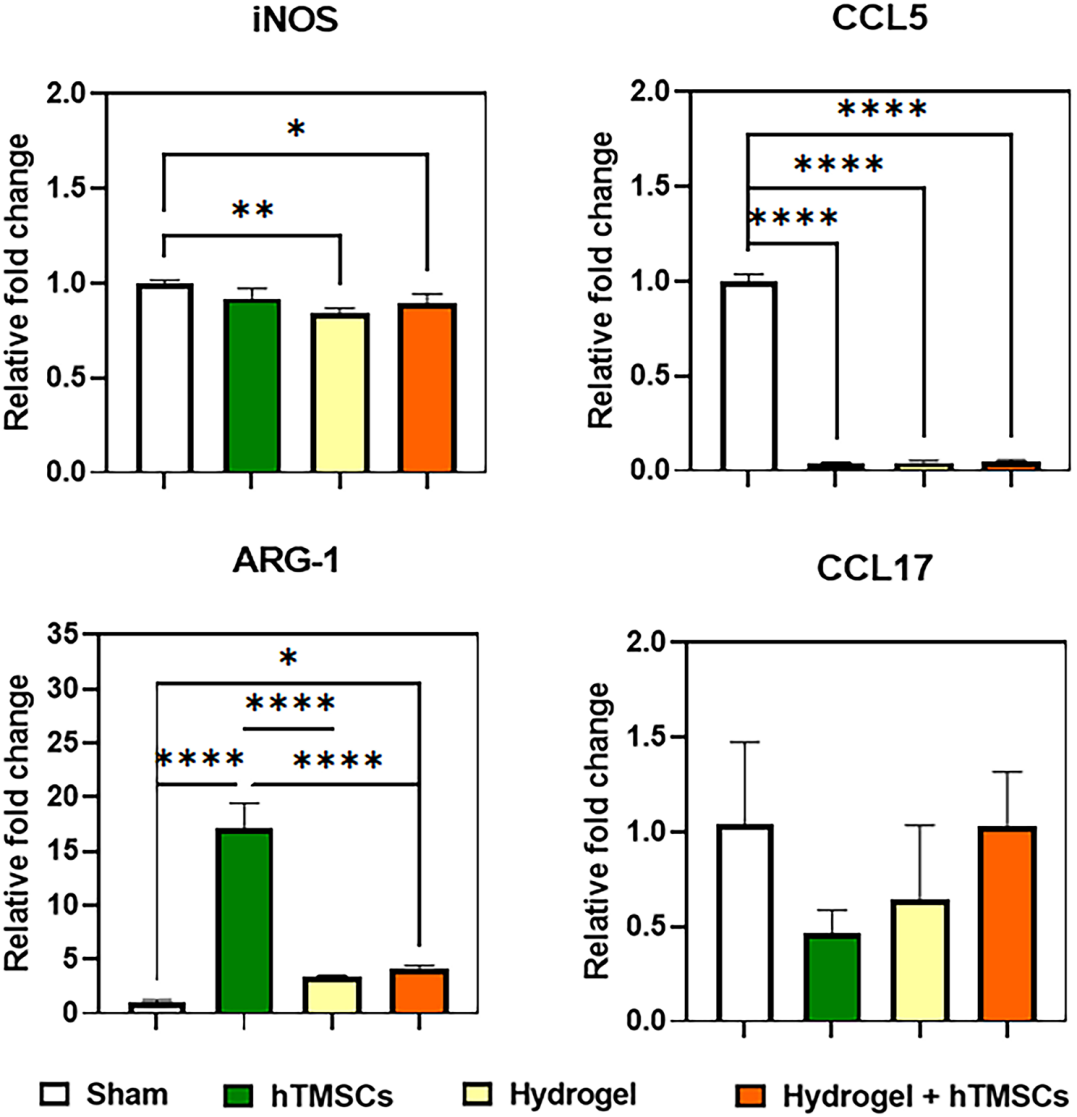
Analysis of the anti-inflammation effects of macrophage polarization using RT-PCR. The anti-inflammatory effects were analyzed using macrophage polarization markers through RT-PCR in control, hTMSCs, Hydrogel, and the combined treatment group (Hydrogel + hTMSCs). iNOS and CCL5 were used as markers for M1, which is a pro-inflammatory cytokine, while ARG-1 and CCL17 were used as markers for M2, which is an anti-inflammatory cytokine. P values are indicated as *P < 0.05, **P < 0.01, and ****P < 0.0001 by One-way ANOVA-Tukey’s multiple comparisons test (n = 3)

After culturing macrophages using a transwell, they were stimulated with LPS, and each sample was injected into the bottom to assess the macrophages’ polarization degree and confirm the anti-inflammatory effect. The degree of polarization was evaluated using RT-PCR, with iNOS and CCL5 serving as M1 markers, and ARG-1 and CCL17 serving as M2 markers. PCR analysis revealed that the Hydrogel + hTMSCs group and the hydrogel group showed significantly lower expression of iNOS, an M1 marker, relative to the control group. Moreover, ARG-1, an M2 marker, exhibited significantly higher expression in all experimental groups compared to the control group (Fig. 4). The results for CCL5 expression showed a significant decrease in all groups compared to the sham group, and CCL17 levels were elevated in the combined treatment group compared to other treatment groups. However, it is essential to note that the difference observed did not reach statistical significance. These results confirmed that each treatment group displayed an anti-inflammatory effect.

### Macroscopic image analysis of oral ulcer recovery

**Fig. 5:**
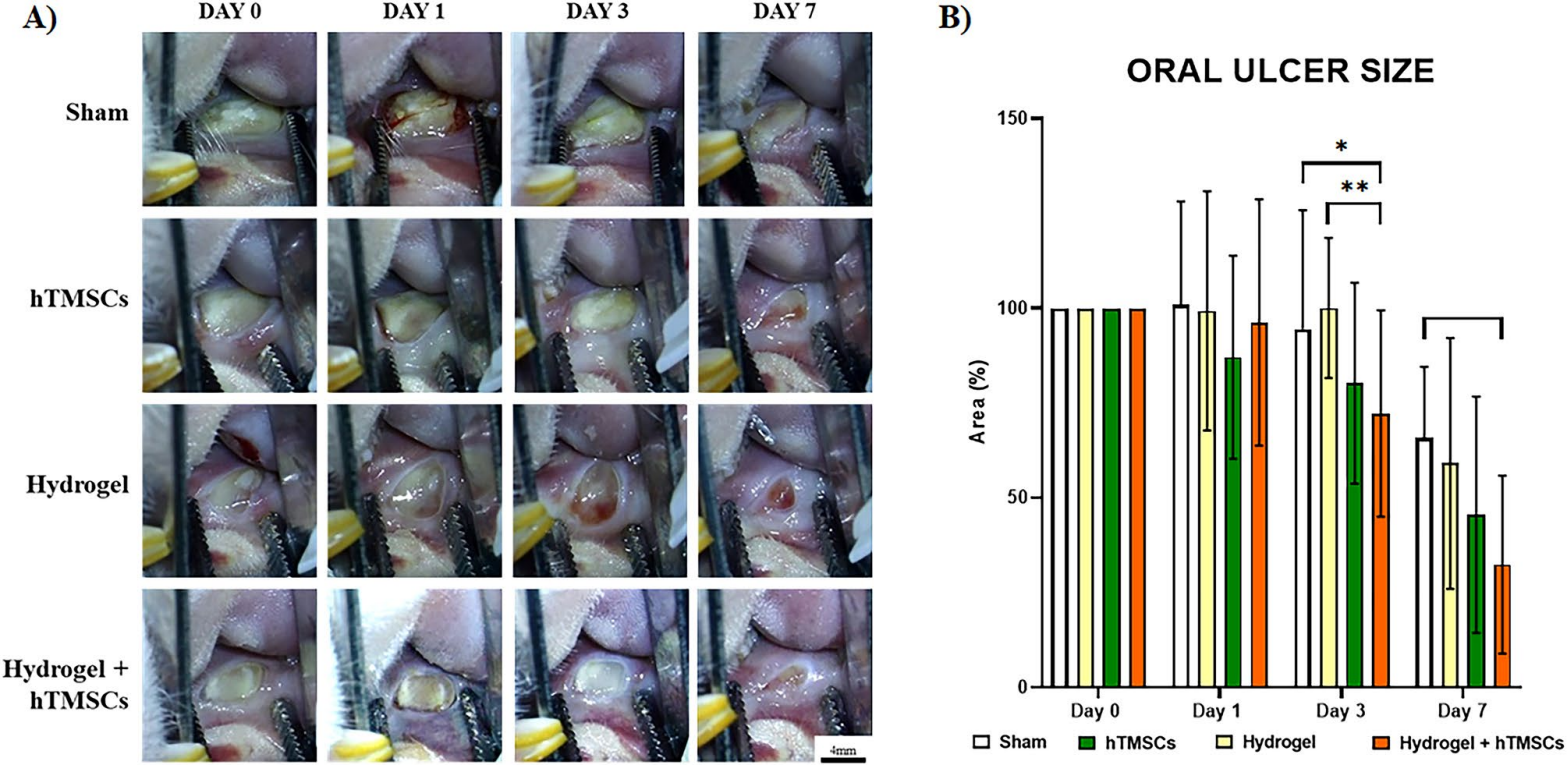
In vivo oral ulcer image and ulcer size measurement results. (**A**) The size of ulcers in chemically induced rats was measured on Day 0, Day 1, Day 3, and Day 7. (**B**) Percentage of the residual ulcer area in hTMSCs, Hydrogel, Hydrogel + hTMSCs, and sham groups on Day 0, Day 1, Day 3, and Day 7. All image analyses have been performed using ImageJ software. P values are indicated as *P ≤ 0.05 and **P ≤ 0.01 by two-way ANOVA-Turkey’s multiple comparisons test (number of images analyzed per sample: n = 8)

It was confirmed that the model was created by checking the presence or absence of ulcers on Day 3 after induction of ulcers. Oral ulcers were photographed on Days 0, Day 1, Day 3, and Day 7 (Fig. 5A). Figure 5B compares the rate of ulcer size reduction to the initial size. The sham group exhibited the lowest reduction rate compared to the other experimental groups at all observation points. On Day 3, the Hydrogel + hTMSCs group demonstrated a significant decrease in size in comparison to the sham (P < 0.05) and Hydrogel groups (P < 0.001). The Hydrogel + hTMSCs group exhibited the highest reduction on the 7th day, with an average decrease to 32.38% relative to the initial size, and it significantly decreased in size compared to the sham group (P < 0.05, n = 8 images per group). The stem cell group decreased to 45.57%, and the Hydrogel group decreased to 59.13%.

### Histological assessments

**Fig. 6:**
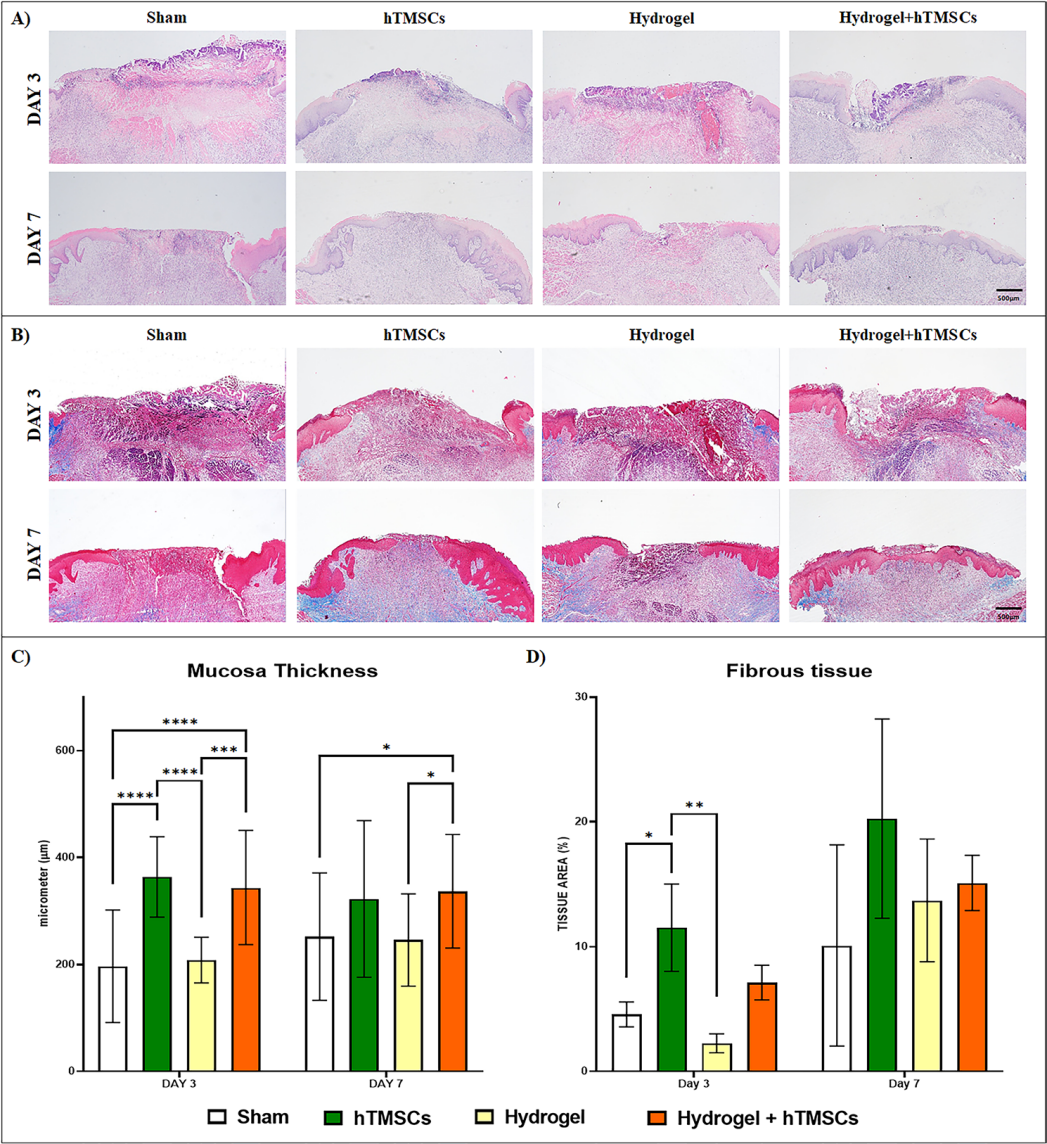
Histological assessment of mucosa thickness and fibrous tissue. (**A**) Hematoxylin and eosin staining of the wound on Day 3 and Day 7 in the sham group and groups treated with hTMSCs, Hydrogel, and Hydrogel + hTMSCs. Note that by Day 7 complete re-epithelialization and granulation tissue were apparent in the Hydrogel + hTMSCs group. (**B**) Histological assessment of fibrous tissue in tissue sections stained with MT. Collagen was observed in the areas where the ulcers formed in the hTMSCs and Hydrogel + hTMSCs groups on Day 7 (scale bar = 500 μm). (**C**) Measurement of the mucosal layer thickness indicates that oral ulcers treated with the hTMSCs and Hydrogel + hTMSCs were the thickest on Day 3. Only the group treated with Hydrogel + hTMSCs showed a significant difference from the Hydrogel and sham group on Day 7. (**D**) Quantified fibrous tissue (blue stained collagenous tissue) in all groups on Days 3 and 7 based on image analysis of Masson’s trichrome-stained sections. On Day 3, the stem cell treatment yielded the most fibrous tissue formation compared to the sham and the Hydrogel groups. By Day 7, the stem cell-treated group still showed higher fibrous tissue formation but was no longer significantly better than all other groups. P values are indicated as *P < 0.05, *P < 0.05, and **P < 0.01 by one-way ANOVA-Tukey’s multiple comparisons test (n = 4)

Histological analyses of wound regeneration were conducted using H&E and MT staining. H&E staining confirmed the ulcer’s depth and inflammatory cells’ presence (Fig. 6A). The mucosal membrane thickness was significantly greater in the hTMSCs and Hydrogel + hTMSCs groups compared to the sham group. No significant difference was observed between the sham and Hydrogel groups (Fig. 6C). MT staining was employed to visualize collagen fibers and fibrous tissue. A substantial amount of collagen fibers was observed beneath the ulcer interface in the hTMSCs group and the hydrogel + hTMSCs group (Fig. 6B). The area of fibrous tissue increased in all groups over time without any significant difference (Fig. 6D).

**Fig. 7:**
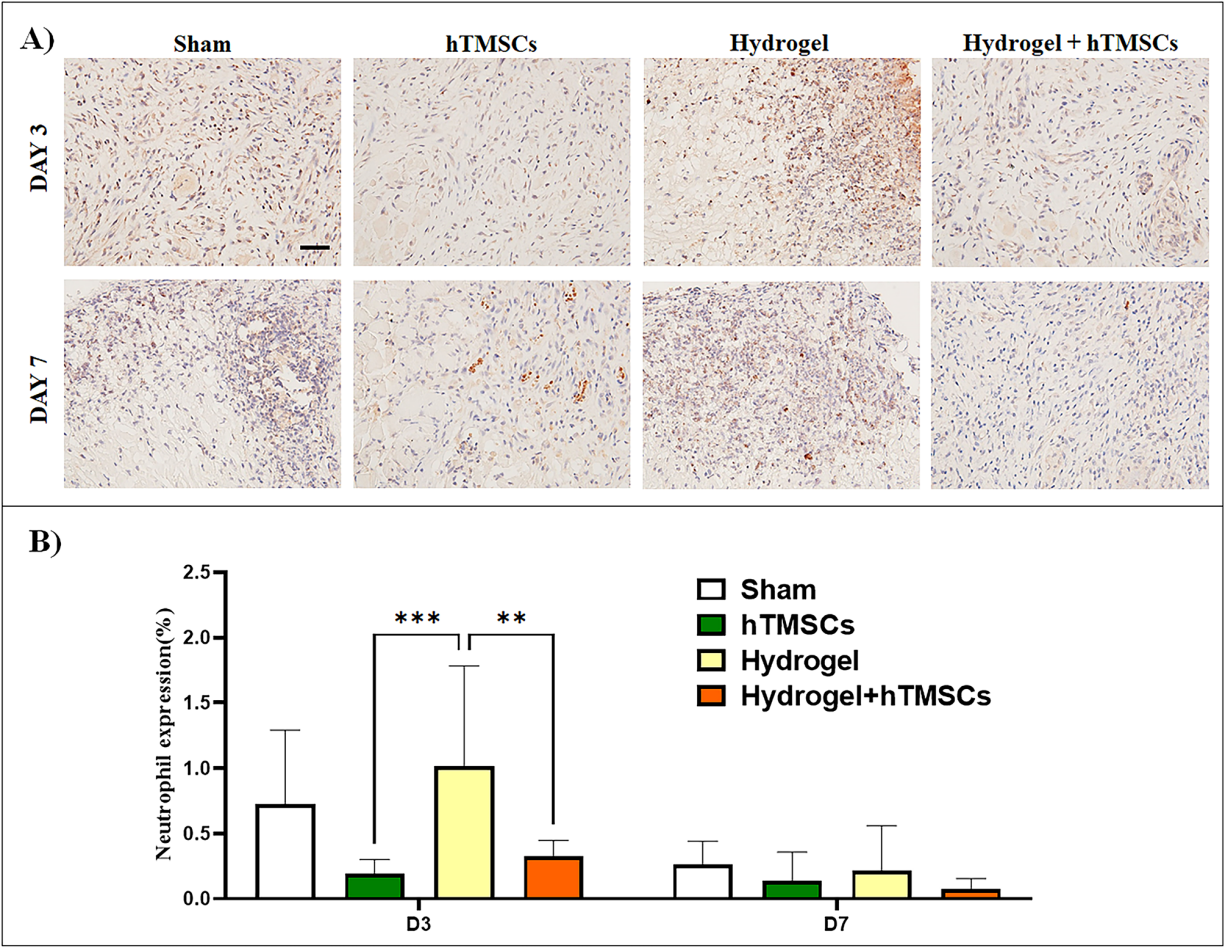
Confirmation of neutrophils expression to confirm inflammatory response. (**A**) IHC Staining for the measurement of neutrophil expression (Scale bar = 50µm). (**B**) Quantified neutrophil elastase expression in tissue sections based on image analyses. The Hydrogel-treated group showed significantly high neutrophil expression compared to the hTMSCs and Hydrogel + hTMSCs treated groups. No difference among groups was observed by Day 7. P values are indicated as *P < 0.05 by one-way ANOVA-Turkey’s multiple comparisons tests (number of images analyzed: n = 7)

IHC staining of neutrophils was performed to confirm the presence of inflammatory cells (Fig. 7A). The ulcer area was photographed and compared. On Day 3, it was most frequently observed in the hydrogel group; on Day 7, it was most frequently observed in the sham and Hydrogel group. The presence of neutrophils within the tissue samples from each treatment group was quantified via image analysis of the IHC-stained tissue sections. The results indicated that on day 3 post-treatment, the Hydrogel group contained significantly higher amounts of neutrophils than the stem cell and combined treatment groups. On Day 7, there was no significant difference among group treatments, with the combined treatment group showing the least neutrophil (Fig. 7B).

**Fig. 8:**
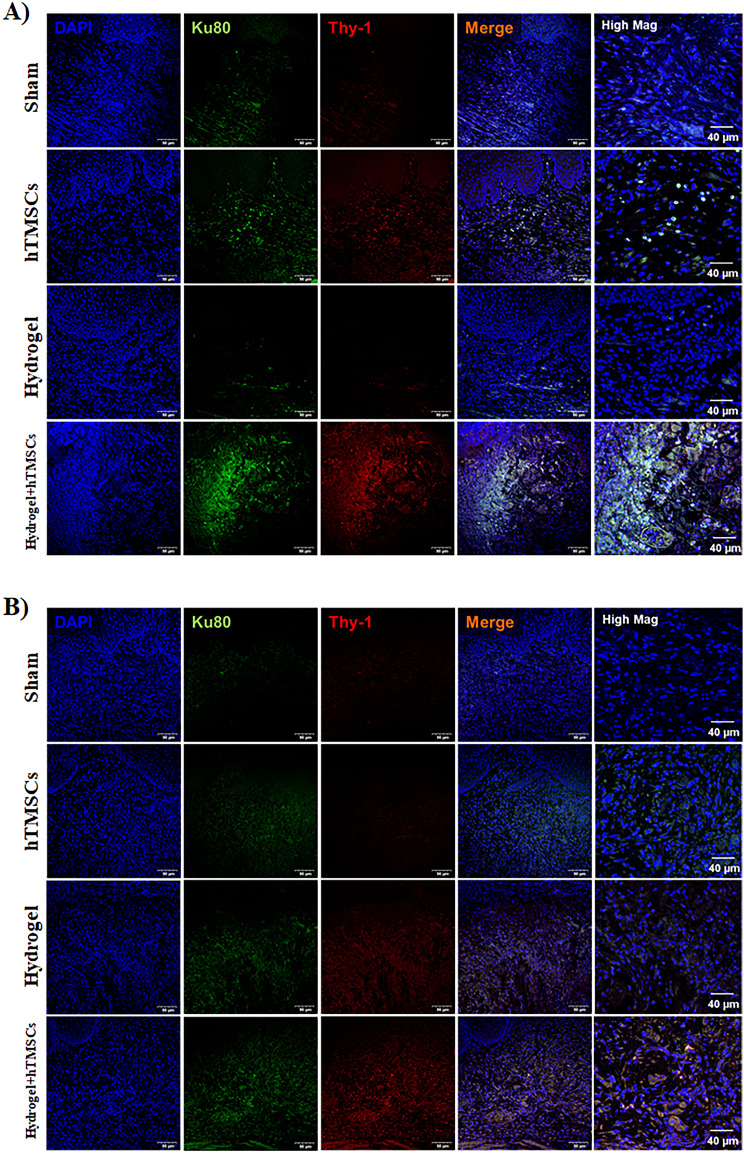
Immunofluorescence staining for verifying the survival and retention of stemness of transplanted stem cells in the stem cell and combined treatment groups. (**A**) On the 3rd day, the presence of transplanted stem cells was confirmed in both the hTMSCs group and the hydrogel + hTMSCs group. (**B**) Only cells in the hydrogel + hTMSCs group retained stem cell markers. The difference was prominently displayed in the high-magnification merged image showing the co-localized expression of both Ku80 and Thy-1 markers

Immunofluorescence staining was performed to verify the survival and retention of stemness of transplanted stem cells in the stem cell and combined treatment groups. This was achieved by utilizing Ku80 (green) as a marker for human-derived cells and Thy-1 (red) as a marker for mesenchymal stem cells. On Day 3, the presence of transplanted stem cells was confirmed in both the hTMSCs group and the Hydrogel + hTMSCs group (Fig. 8A). However, only cells in the Hydrogel + hTMSCs group retained stem cell markers by Day 7. The difference was prominently displayed in the high-magnification merged image showing the co-localized expression of both Ku80 and Thy-1 markers (Fig. 8B).

### The inflammatory response is based on mRNA expression levels

**Fig. 9:**
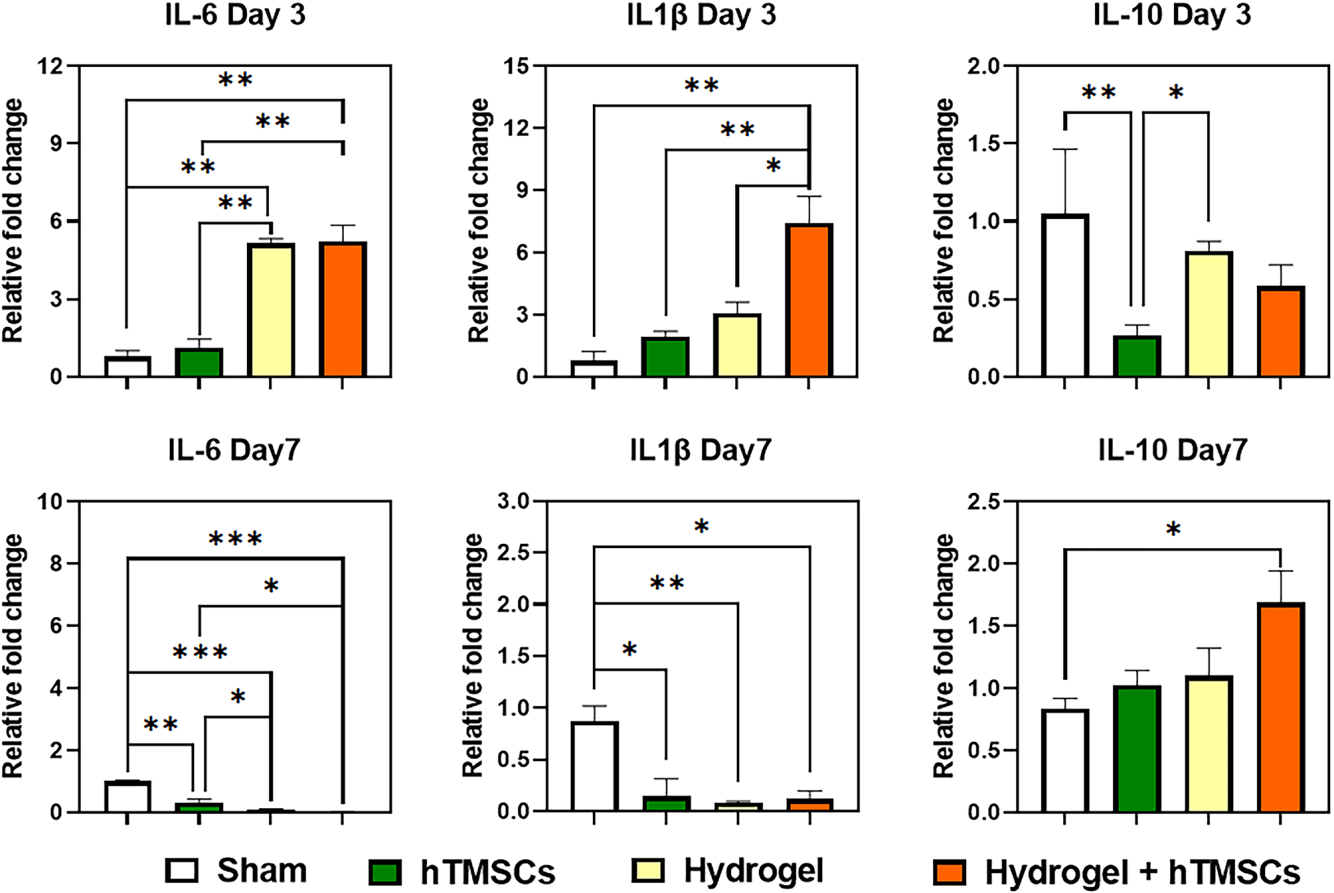
Inflammatory cytokine expression in rat’s buccal tissue. The mRNA expression of IL-6, IL-1β, and IL-10 was examined by RT-qPCR and normalized to GAPDH. P values are indicated as *P ≤ 0.05, **P ≤ 0.001, and ***P ≤ 0.0002. by one-way ANOVA-Turkey’s multiple comparisons tests (number of readings per sample = 10)

The ulcer healing was confirmed by measuring the expression levels of inflammatory cytokines in the tissues. Interleukin (IL)-6 and IL-1β were used as pro-inflammatory cytokines, while IL-10 was used as an anti-inflammatory cytokine (Fig. 9). Regarding the expression level of IL-6, it was observed that both hydrogel-administered groups showed a significant increase on Day 3 compared to the other groups. However, on Day 7, it was found that all treatment groups exhibited a significant decrease in IL-6 expression compared to the sham group. In the case of IL-1β, the combined treatment group displayed the highest expression on Day 3, with a significant difference compared to the other groups. On Day 7, it was observed that IL-1β expression decreased compared to the sham group.

Regarding IL-10, an anti-inflammatory cytokine, the sham group exhibited the highest level on Day 3. However, on Day 7, the combined treatment group demonstrated the highest level of IL-10 expression, with a significant difference compared to the sham group.

### Immunohistochemical analysis of anti-inflammatory cytokine in oral ulcers

**Fig. 10:**
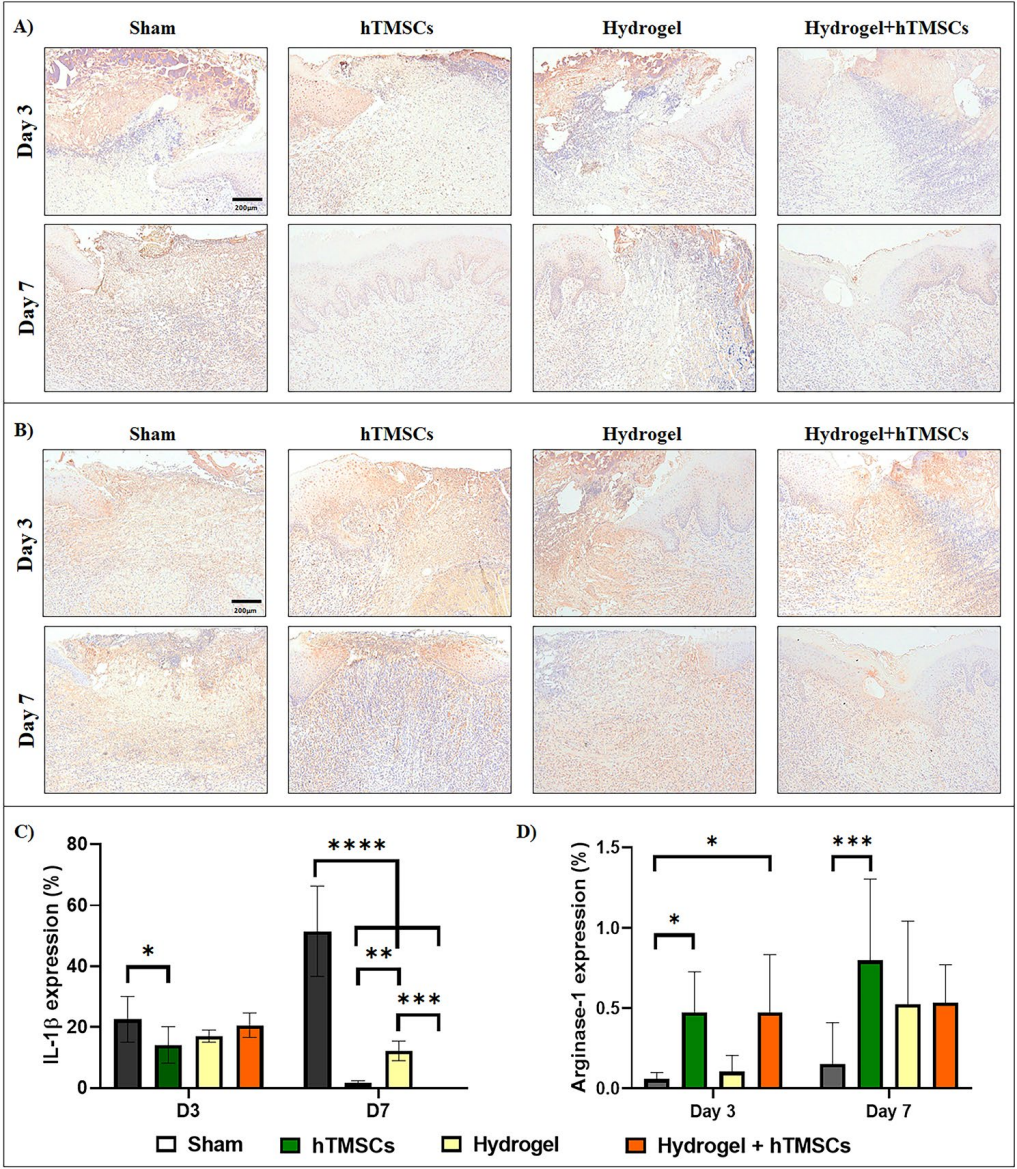
Immunohistochemical analysis of cytokines for identification of inflammatory response in oral ulcer. (**A**) Representative microscopic image of IL-1β. (**B**) Representative microscopic image of ARG-1. (**C**) Comparative analysis of quantification data of positively stained regions in IHC staining for IL-1β. The sham group consistently showed higher IL-1β on Day 3 and Day 7. Conversely, the group treated with hTMSCs and Hydrogel + hTMSCs showed consistently lowered expression of IL-1β, particularly on Day 7. (**D**) Comparative analysis of quantification data of positively stained regions in IHC staining for ARG-1. On Day 3, the groups treated with hTMSCs were higher compared to both sham and Hydrogel-treated groups. On Day 7, only the hTMSCs-treated group showed significantly higher expression of ARG-1 compared to the sham group. P values are *P ≤ 0.05 and **P ≤ 0.01. by one-way ANOVA-Turkey’s multiple comparisons tests (n = 3)

Immunohistochemical staining was performed to compare the inflammatory response associated with recovery. On day 3, IL-1β, a pro-inflammatory cytokine, was prominently expressed in the Hydrogel group. By the 7th day, the sham group exhibited the highest level of IL-1β expression, while the Hydrogel group still showed signs of an ongoing inflammatory response (Fig. 10A).

Quantitative results of IL-1β staining showed low levels overall in the experimental group compared to the sham group on day 3. In particular, significant differences were confirmed only in the hTMSCs group. In the results of day 7, the sham group increased, and all other experimental groups showed significant differences compared to the sham group. However, it was confirmed that the IL-1β expression was still high in the hydrogel group (Fig. 10C).

Regarding ARG-1 staining, which is related to recovery, it was observed that M2 macrophages were positively stained in the stem cell and combined treatment groups starting Day 3. On Day 7, the staining intensity was the strongest in the stem cell group and the weakest in the sham group, indicating a greater presence of M2 macrophages in the stem cell group during the recovery process (Fig. 10B).

Quantitative analysis of ARG-1 found significant differences in the hTMSCs group and the mixed treatment group compared to the sham group on Day 3 and significant differences compared to the sham group only in the hTMSCs group on Day 7 (Fig. 10D).

## Discussion

Oral mucositis, a well-known adverse effect of chemotherapy, has posed a persistent challenge in cancer treatment [34, 35]. Despite numerous attempts, only a few treatments have demonstrated efficacy in managing oral mucositis. Recently, a growing body of research has focused on stem cell and hydrogel-based interventions for oral mucositis [8, 18]. Stem cell therapy has shown promising outcomes in tissue regeneration employing paracrine signaling and secretion. MSCs have attracted significant attention, and research in this area has experienced exponential growth. MSCs possess the capacity for self-renewal and can differentiate into multiple cell types. They play crucial roles in tissue healing and regenerative medicine. MSCs originate from the mesoderm during embryonic development and are abundant in tissues like the umbilical cord, bone marrow, and adipose tissue. These cells possess remarkable regenerative and differentiation abilities and have garnered significant interest and research attention concerning various diseases, including neurological diseases, blood disorders, and inflammatory conditions [36–38]. This interest arises because MSCs exhibit a range of functions, such as differentiation, antioxidant properties, anti-apoptotic effects, anti-inflammatory effects, and promotion of cell regeneration [39–45]. However, maintaining viable cells within the oral mucosa presents challenges due to proteolytic enzyme damage during cell preparation and disruption during feeding processes [46]. In this study, the hydrogel was loaded with stem cells and administered via local injection as a treatment strategy to overcome challenges associated with cell retention and viability.

For the stem cells, we opted to utilize excised human tonsil tissues rather than the more commonly used bone marrow-derived stem cells. Extraction of stem cells from bone marrow poses challenges due to invasive procedures, low yields, and limited proliferation rates. Thus, new studies are underway, focusing on alternative sources such as tonsil-derived mesenchymal stem cells. Tonsil tissue has gained attention as an attractive resource because it can be obtained from discarded tissue after tonsillectomy, eliminating unnecessary surgeries [20, 47, 48]. In addition, there are various advantages, such as higher yield than MSCs derived from bone marrow and fat, long-term passage culture, and preservation [49, 50]. Various studies have shown stem cell therapy’s successful role in treating oral mucosal lesions, such as oral ulcers and premalignant conditions [51, 52]. Jung et al., reported the therapeutic effects of tonsil-derived MSCs with matrigel in a hamster model of OM induced by 5-fluorouracil (FU) [17]. Tonsil-derived mesenchymal stem cells were isolated from tonsil tissue from a patient who underwent a tonsillectomy. A cell flow analyzer confirmed the presence of mesenchymal stem cell-specific markers CD90, CD105, and CD34. According to previous studies by Lee et al. and Choi et al., CD90 showed a presence of more than 98%, CD105 more than 99.9%, and CD34, a marker specific to hematopoietic cells, was not detected [53, 54]. Furthermore, the morphological properties of the isolated mesenchymal stem cells were observed, as described in the study by Haaster et al., confirming the successful isolation and cultivation of tonsil-derived mesenchymal stem cells [55].

Cytotoxicity testing was performed using the same concentration of TMC as used in previous studies, demonstrating no toxicity at or below 5%. TMC is a desirable and multifunctional polymer with a positive charge due to its quaternized nature. Chitosan hydrogel, being biodegradable and biocompatible, offers the advantage of not requiring removal post-implantation, unlike other implanted materials [56, 57]. This characteristic enables it to exhibit functions such as drug delivery, antimicrobial activity, anti-adhesive properties, hemostasis, and wound healing [28]. By adding a naturally occurring compound called β-gp, gelation occurs through sol-gel transition at physiological pH and 37℃. At room temperature, the hydrogel is liquid and can be injected into the body using a syringe. Upon injection, the liquid hydrogel transforms into a degradable gel, which can be used for drug delivery or as a cell carrier [29, 30]. Upon optimization of the hydrogel formulations, the toxicity of TMC hydrogel toward stem cells and its suitability for transplantation were assessed. Previous studies have demonstrated that the porous structure of the hydrogel is typically maintained for 7 days in vitro but undergoes significant degradation in vivo [58]. Based on the hydrogel degradation test, fragmentation of the solidified TMC hydrogel was observed within 5 days of incubation in vitro. This demonstrated that the solidified TMC hydrogel can gradually release the encapsulated stem cells into a tissue-like environment over time.

Current stem cell therapies are often challenged with cell attachment failure due to the use of proteolytic enzymes during the stem cell preparation process [46]. In this study, we aimed to address this issue by utilizing a TMC hydrogel mixture as a scaffold. Evaluating Ku80 staining demonstrated that the experimental group treated solely with stem cells can be observed on Day 3 but is no longer present on Day 7. Conversely, when stem cells were delivered with the thermosetting TMC hydrogel as a treatment, it persisted until day 7. Previous research by Liu et al. reported that local injection of MSCs reduced CD31 expression and suppressed IL-6 levels in an animal model [59]. Analysis of the PCR results of the tissue samples revealed that on the 7th day, the hydrogel + hTMSCs group exhibited a significant decrease in expression compared to the group treated with stem cells alone. Compared to the control-sham group, this decrease confirmed the longer retention of the stem cells.

Additionally, previous studies by Abueva et al. [60] have reported the anti-inflammatory properties of TMC. It has been documented that implanted biomaterials can elicit an immune response [61]. As illustrated by the initial tests involving classically polarized macrophage in vitro, the stem cell exerted a substantially greater extent of immune modulation compared to the lone TMC hydrogel while the combination of the TMC hydrogel and hTMSCs exhibited relatively similar results to a lower extent when compared to the control. Consistent with these findings, the TMC hydrogel and combined treatment group exhibited a heightened inflammatory response compared to the control group on the third day following initial transplantation. However, analysis of the results on day 7 revealed a decreasing trend in inflammatory response. This reduction can be attributed to the combined anti-inflammatory effect of the TMC and the relative tissue response to the transplanted stem cells. Despite the observed degradability and fragmentation of the TMC hydrogel, the combined effect was apparent until the last observation period.

Although the stem cell used for this study was sourced from human tonsil tissues, it did not elicit adverse immunological responses in the test animals. More so examination of tissue samples stained with hematoxylin and eosin and MT stain confirmed the improved early tissue mucosal tissue regeneration and relatively lower fibrosis in hTMSCs injected with TMC hydrogel compared to the direct injection of hTMSCs. Mesenchymal stem cells are hypoimmunogenic in humans and animals. This is taken into account for three main reasons. First, mesenchymal stem cells don’t provoke a strong immune response because they often lack certain immune system markers like MHC-II and other costimulatory molecules. Second, these stem cells hinder T cell responses indirectly by affecting other immune cells like dendritic cells. Third, mesenchymal stem cells create a suppressive environment around them by producing certain substances like L-10 and prostaglandins that regulate the immune response [62]. Previous studies have indicated that stem cells play a key role in fibrosis in different tissues and fibrotic disorders [63, 64]. Infusions of stem cells in mice have been observed to protect from lung fibrosis caused by bleomycin [65], decrease carbon tetrachloride-induced liver fibrosis [66], and even reduce myocardial infarct-related cardiac fibrosis [67]. The relative effect of stem cell infusion was related to the reduction of inflammation, modulation of collagen deposition, and decreased matrix metalloproteinase signaling [65–67]. It should be noted that although the histomorphometric measurement of the fibrous tissue indicates that the direct injection of stem cells induces sustained fibrogenesis, this cannot be attributed to the stem cell since the test regarding cell retention showed significantly low positivity for both markers of human-derived cells and mesenchymal stem cell marker.

Concerning the immunomodulatory effect of stem cells, it should be noted that among the treatments, TMC hydrogel with hTMSCs resulted in increased initial mRNA expression of both inflammatory cytokine (IL-6 and IL-1β) and anti-inflammatory cytokines (IL-10) but eventually sustaining only IL-10 by the end of the in vivo experiment. At the cell level, IL-10 can dampen human antigen R leading to the breakdown of mRNA for inflammatory cytokines. It is also able to inhibit certain cell death pathways like the p38 MAPK pathway, through a signaling process involving STAT3 preventing tissue damage and organ dysfunction following an injury [68–70]. This effect was exemplified upon observing the downregulation of IL-1β expression and sustained Arginase 1 expression in oral ulcers treated with TMC hydrogels and stem cells. Under these conditions, it is expected to considerably influence the resulting regenerated tissue.

Although the efficacy of the TMC-hydrogel with hTMSCs treatment has been confirmed, there are several limitations in this study. Notably, is that this long-short-term follow-up observation is insufficient and the treatment effect has been confirmed, but there is a limitation in that the mechanism cannot be explained with regards to the effect of the hTMSc on mucosal regeneration is not fully elucidated. Nevertheless, this study demonstrates the potential of utilizing an alternative source of mesenchymal stem cells with a chitosan-based thermogeling hydrogel for the treatment of oral ulcers.

## Conclusions

This study highlights the potential of resolving adhesion and viability issues in current stem cell therapies by employing a hydrogel formulated with Trimethyl Chitosan (TMC), a biomaterial, and combining it with tonsil-derived mesenchymal stem cells. The synergistic effect of this combined treatment demonstrates the capability to accelerate the healing of oral ulcers. This is achieved through the augmentation of anti-inflammatory effects and the facilitation of wound healing, effectively reducing inflammatory responses and extending the survival of transplanted stem cells. However, it is essential to acknowledge that further research is necessary to minimize the initial inflammatory response of biomaterials. Moreover, a comprehensive assessment of safety and long-term effects is crucial for evaluating the viability of this approach in potential clinical applications. This study lays a foundation for future investigations aimed at refining these promising therapies for enhanced clinical outcomes.

### Electronic supplementary material

Below is the link to the electronic supplementary material.

Supplementary Material 1

Supplementary Material 2

## Data Availability

The datasets during and/or analysed during the current study available from the corresponding author on reasonable request.

## Abbreviations

TMC: N,N,N-trimethyl chitosan/ trimethyl chitosan
PBS: phosphate-buffered saline
BSA: bovine serum albumin
DPBS: Dulbecco’s phosphate-buffered saline
hTMSCs: human tonsil-derived mesenchymal stem cells
MSCs: mesenchymal stem cells
iPS: induced pluripotent stem cells
H&E: hematoxylin and eosin
MT: Masson’s trichrome
β-gp: beta glycerophosphate
IL: Interleukin
