# Supplementary figures and images for: Oral ulcer treatment using human tonsil-derived mesenchymal stem cells encapsulated in trimethyl chitosan hydrogel: an animal model study
Source: Stem Cell Res Ther. 2024 Apr 8;15:103. doi: 10.1186/s13287-024-03694-4 (PMC11003084; doi:10.1186/s13287-024-03694-4)

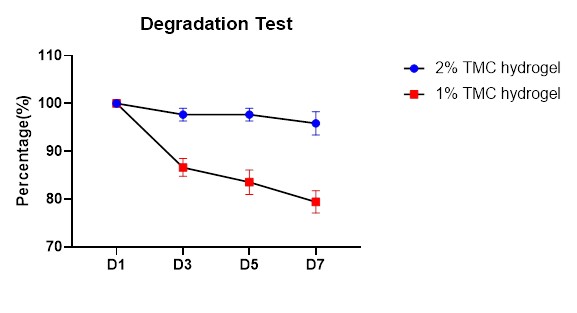

Supplement: Supplementary file 1 — Supplementary Material 1 [file 13287_2024_3694_MOESM1_ESM.jpg]

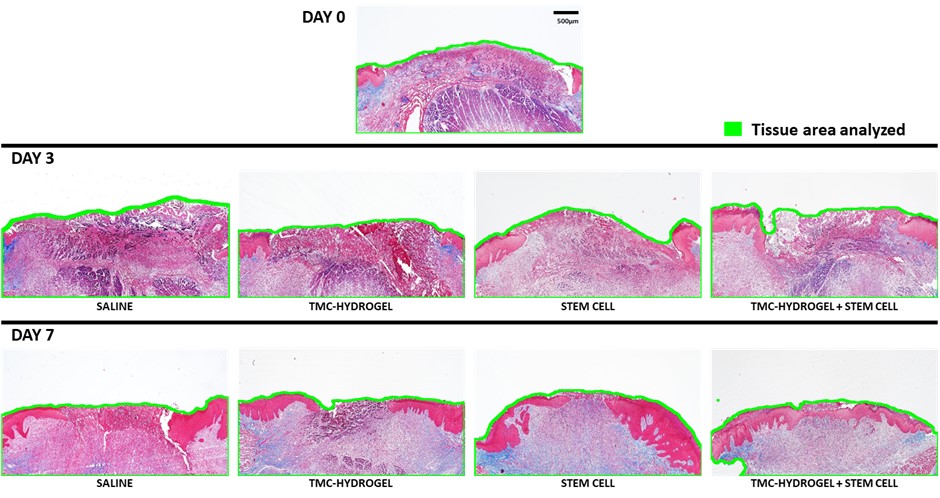

Supplement: Supplementary file 2 — Supplementary Material 2 [file 13287_2024_3694_MOESM2_ESM.jpg]
